# Diagnostic Value of Cardiovascular Magnetic Resonance T1 and T2 Mapping in Acute Myocarditis: A Systematic Literature Review

**DOI:** 10.3390/medicina60071162

**Published:** 2024-07-18

**Authors:** Karolina Gaizauskiene, Kamile Leketaite, Sigita Glaveckaite, Nomeda Valeviciene

**Affiliations:** 1 Department of Radiology, Nuclear Medicine and Medical Physics, Institute of Biomedical Sciences, Faculty of Medicine, Vilnius University, 03101 Vilnius, Lithuania; nomeda.valeviciene@santa.lt; 2 Faculty of Medicine, Vilnius University, 03101 Vilnius, Lithuania; kamile.leketaite@mf.stud.vu.lt; 3 Clinic of Cardiac and Vascular Diseases, Institute of Clinical Medicine, Faculty of Medicine, Vilnius University, 03101 Vilnius, Lithuania; sigita.glaveckaite@santa.lt

**Keywords:** myocarditis, cardiac magnetic resonance, diagnostic value, mapping, T1 mapping, T2 mapping

## Abstract

*Background and Objectives*: Over the past decade, there has been increasing attention paid to advanced and innovative cardiovascular magnetic resonance (CMR) modalities, such as T1 and T2 mapping, which play a major role in diagnosing diffuse myocardial disease. There is little data summarizing the current evidence regarding the diagnostic accuracy of T1 and T2 mapping, and extracellular volume (ECV) in acute myocarditis. The aim of our study was to select, analyze, and systematically review the recent scientific literature on the diagnostic value of CMR T1 and T2 parametric mapping in clinically suspected acute myocarditis. *Materials and Methods*: The literature search was performed in the PubMed database. Articles published in the years 2014–2024 were included in the analysis. At the initial stage, 458 articles were reviewed, and 13 exploratory research studies were further analyzed and presented in this systematic literature review. *Results*: The analysis included 686 patients with clinically suspected myocarditis and 372 subjects in the control group. The average age of patients with suspected myocarditis was 40.25 years; 26% of them were women. Prolonged native myocardial T1 relaxation time provides diagnostic accuracy in the setting of suspected acute myocarditis ranging from 69 to 99%, with sensitivity from 64 to 98% and specificity from 87 to 100%. Diagnostic accuracy of prolonged T2 relaxation time ranges from 47 to 87%, with sensitivity being from 48% to 94% and specificity from 60% to 92%. ECV alone showed moderate diagnostic performance, with diagnostic accuracy ranging from 62% to 76%, sensitivity from 47% to 73%, and specificity from 76% to 90%. T1 and T2 mapping and ECV, combined with the late gadolinium enhancement (LGE) technique, increases the probability of detecting myocardial inflammatory changes at various stages of the disease, improving the diagnostic accuracy to 96%. *Conclusions*: New quantitative CMR techniques, i.e., T1 and T2 mapping, have an advantage over conventional CMR sequences in detecting inflammatory myocardial structural changes and play an important role in diagnosing acute myocarditis. Incorporating these sequences in daily clinical practice increases the diagnostic value of CMR in acute myocarditis and becomes an alternative to endomyocardial biopsy, which has been considered the gold standard until now.

## 1. Introduction

Acute myocarditis is an inflammatory disease of the heart muscle, with a recent onset, typically occurring within one month. It can be caused by different infectious agents like viruses and bacteria, along with exposure to medications, toxins, and hypersensitivity reactions. In developed countries, lymphocytic myocarditis, mainly induced by viral pathogens, is the most common cause [1,2]. It is estimated that 1% to 5% of patients with acute viral infections develop myocardial inflammation [3].

The clinical presentation of myocarditis can vary greatly, including asymptomatic cases, generalized fatigue, chest pain, dyspnoea with acute coronary syndrome-like presentation, arrhythmias, cardiogenic shock, chronic heart failure, and sudden cardiac death [4]. Although patients with mild symptoms and uncomplicated myocarditis usually recover without specific treatment, the disease is considered a major cause of sudden cardiac death in young active adults. Research indicates a wide range of autopsy findings of myocardial inflammation in young individuals who suffer sudden cardiac death, varying from 2% to 42%. Additionally, up to 30% of cases of biopsy-proven myocarditis may progress to dilated cardiomyopathy with a poor prognosis [5].

Globally, myocarditis is estimated to occur in 1.8 million cases annually [6]. However, due to its diverse clinical picture and challenges in clinical diagnosis, myocarditis is considered underdiagnosed, and its exact incidence is unknown. Moreover, studies during the pandemic have shown that COVID-19 has led to an increase in cases of acute myocardial injury, including acute myocarditis. This highlights the importance of early recognition and management of the condition [7].

Endomyocardial biopsy (EMB) is the gold standard method for diagnosing acute myocarditis; however, given its invasiveness, potential complications, and lack of sensitivity and specificity, it is not routinely used in daily practice [2,5].

In this context, CMR has progressed significantly and has emerged as the most potential non-invasive imaging tool for diagnosing and managing inflammatory heart diseases. Since 2009, the Lake Louise criteria (LLC) has been used to diagnose myocarditis by CMR. It targets three aspects of myocardial inflammation, including edema, hyperaemia, and necrosis or fibrosis. Image interpretation of the original LLC relied on analysis of signal intensities on T2-weighted, early gadolinium enhancement (EGE), and late gadolinium enhancement (LGE) images [8,9]. Over the past decade, there has been increasing attention on advanced and innovative CMR modalities, such as T1 and T2 mapping. There are many disadvantages of conventional CMR sequences. Conventional CMR methods rely on their qualitative or semiquantitative data, allowing only a comparative analysis between normal and diseased myocardium, which cannot be compared among subjects or in dynamics. Limitations of LGE: incomplete myocardium nulling, insensitive to detecting diffuse interstitial fibrosis, sensitive to motion artifacts, and does not differentiate well between acute and chronic myocardial injury. The limitations of T1-weighted or T2-weighted imaging are artifacts resulting from extended acquisition times and artifacts leading to an artificially low signal intensity of the tissue in the case of edema in T2-weighted imaging. T2 short tau inversion recovery (STIR) limitations are incomplete blood suppression, signal dropouts in the lateral wall, and lower signal-to-noise ratios [10].

In 2018, the LLC were updated to include parametric mapping techniques such as T1 and T2 relaxation times and extracellular volume (ECV). The LLC were revised with a requirement to meet both of the following criteria for acute myocarditis: myocardial edema (as assessed by using a global or regional increase in myocardial T2 relaxation time or an increased signal intensity in T2-weighted CMR images) and at least one marker of inflammatory myocardial injury (increased myocardial T1 or ECV or LGE). T1 and T2 mapping techniques have shown promising results in detecting myocardial inflammation, necrosis, or fibrosis, which makes them valuable tools for improving the challenging diagnostic process of myocarditis [11].

This systematic review of the literature provides an overview of the current evidence regarding the diagnostic accuracy of T1 and T2 mapping, as well as ECV, in acute myocarditis, along with the potential implications of parametric mapping for clinical practice in patients with suspected myocarditis.

## 2. Materials and Methods

### 2.1. Data Sources and Search Strategy

This systematic review was conducted using the PRISMA (Preferred Reporting Items for Systematic Reviews and Meta-Analyses) technique. The search was carried out in the PubMed database until 6 March 2024, using the following keywords with MeSH terms. We have used three concepts: myocarditis (“myocarditis”), diagnostic value (“diagnostic value” OR “diagnosis”), and cardiac MRI (“Cardiovascular magnetic resonance” OR “CMR” OR “cardiac magnetic resonance” OR “late gadolinium enhancement” OR “delayed gadolinium enhancement” OR “LGE” OR “mapping” OR “T1” OR “T2” OR “ECV” OR “extracellular volume”). The terms were combined by “OR” in each domain, and then concepts were combined by “AND”.

Search results were imported into Zotero reference management software (Zotero version 6.0.35).

### 2.2. Study Selection

To determine study eligibility, the following inclusion criteria were used:

(1) The study must involve adult patients with clinically suspected acute myocarditis that was diagnosed within 14 days from symptom onset;
(2) CMR must have been performed with either 1.5 T or 3 T field strength machines;
(3) Qualitative or quantitative reporting of at least one CMR parameter of interest, namely LGE, T1 mapping time, T2 mapping time, or ECV;
(4) The study should be written in English;
(5) The study needs to be published within the past decade.

### 2.3. Data Collection

The data were extracted by two independent reviewers (K.G. and K.L.). Information extracted from each publication included the first author’s name, country of origin, type of study, sample size, mean age, gender distribution, field strength, the reference standard for the diagnosis of myocarditis, days between symptom onset and CMR performed, and parameters analyzed in each study. The extracted CMR parameters were LGE, T1 mapping time, T2 mapping time, and ECV.

### 2.4. Study Quality

The QUADAS-2 (Quality Assessment of Diagnostic Accuracy Studies) tool was used to assess the methodological quality of the included diagnostic accuracy studies (Supplementary files Table S1).

## 3. Results

**Figure 1:**
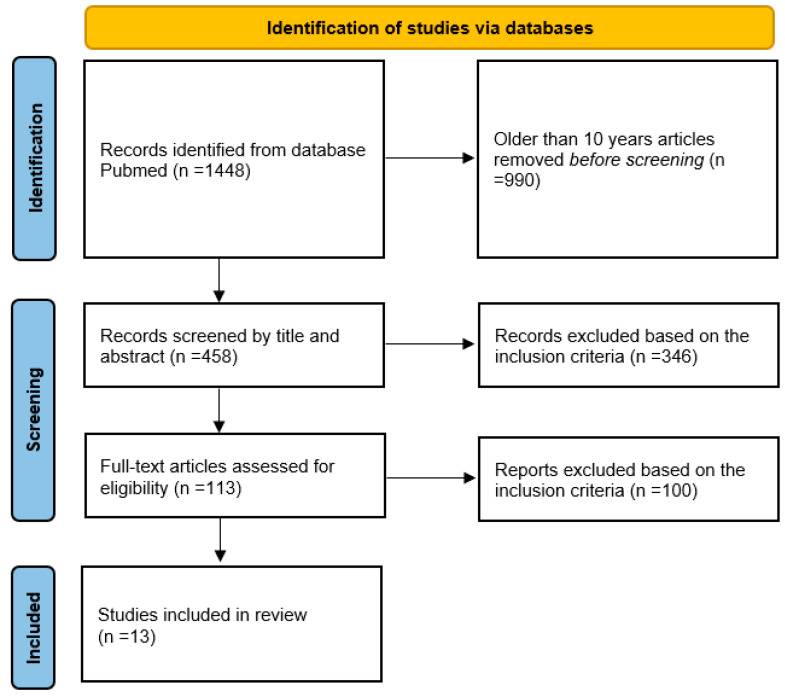
Flowchart of the study.

There were 1448 studies identified through our search, out of which 458 titles and abstracts were retrieved and reviewed for inclusion; 346 were excluded based on the inclusion criteria, and the remaining 113 were screened for eligibility. Finally, 13 studies were included in this systematic review. Reviews, case reports, editorial comments, meta-analyses, and non-English language articles were excluded. The flowchart of study selection is shown in Figure 1. All the studies that were included in the analysis were published between 2014 and 2023; 8 of them were retrospective, while 5 were prospective studies.

The analysis covered a group of 686 patients with myocarditis along with 372 controls. The mean age of the patients with suspected myocarditis was 40.25 years, with 26% being female. The control group had a mean age of 37.46 years, with 24% being female. One study used EMB to confirm the diagnosis of acute myocarditis, while the diagnosis in the remaining 12 studies was based on clinical criteria only.

All studies were performed using a 1.5 T magnet strength, with one study using 3 T. The included studies in the analysis reported CMR findings on LGE (10 studies [12,13,14,15,16,17,18,19,20,21]), T1 mapping (7 studies [14,15,16,17,18,19,22]), T2 mapping (8 studies [13,16,17,18,19,22,23,24]), and ECV (5 studies [16,18,19,22,24]). Characteristics of the included studies are presented in Table 1.

Native T1 mapping. Native T1 mapping, through quantitative tissue characterization of T1 relaxation times, allows the detection of myocardial edema, inflammation, and diffuse fibrosis without the use of gadolinium contrast agents [14,15,24]. Six studies reported the diagnostic accuracy of native T1 mapping, including specificity, sensitivity, and predictive values, as shown in Table 2. The included studies showed that patients with acute myocarditis have a notably higher native myocardial T1 relaxation time compared to the control group, with diagnostic accuracy of T1 mapping ranging from 69 to 99%.

The studies varied in sample size, ranging from 40 to 125 participants, reported sensitivity values from 64% to 98%, and specificity values from 87% to 100%. The cut-off values for T1 mapping varied across studies, with the lowest reported at >980 ms and the highest at >1074 ms.

T2 mapping. T2 mapping provides a quantitative evaluation of the tissue water content, effectively differentiating between focal and global myocardial edema [10]. By closely correlating with the free tissue water content, T2 mapping offers a benefit over T1-based techniques in the diagnosis of myocardial inflammation [24]. Seven studies reported the diagnostic accuracy of T2 mapping time in acute myocarditis. All the analyzed studies have shown that patients with active myocarditis had significantly higher median global myocardial T2 values compared to patients without active myocarditis.

Sensitivity ranged from 48% to 94%, while specificity ranged from 60% to 92%. The percentage of diagnostic accuracy varied between 47% and 87%. The cut-off values for T2 mapping varied across studies, with the lowest reported at >54 ms and the highest at >68 ms. Detailed results regarding predictive values and other diagnostic accuracy metrics are provided in Table 3.

Late Gadolinium Enhancement (LGE). A parameter used in the original LLC detects areas of myocyte necrosis and hyperemia when diagnosing acute myocarditis [12,25]. Additionally, it is a powerful tool for distinguishing between ischemic and non-ischemic etiology of heart diseases because of different patterns of distribution in the myocardium [26]. Eight studies provided information on the diagnostic value of LGE, including the specificity, sensitivity, and predictive values detailed in Table 4. The sensitivity of LGE varied from 52% to 92% across the studies. Specificity values for LGE were consistently high, ranging from 77% to 100%, with most studies reporting specificity values above 90%. Overall diagnostic accuracy of LGE ranged from 62% to 92%, with Schwab et al. [20] reporting the highest accuracy of 92%.

Extracellular Volume (ECV). ECV is calculated using native and post-contrast T1 mapping and is used to assess the cellular and extracellular interstitial matrix compartments. ECV aims to divide the myocardium into two parts: a cellular component and an interstitial component, represented as volume proportions [27]. Five included studies evaluated ECV performance in detecting acute myocarditis, but only three of those provided data on the sensitivity, specificity, and diagnostic accuracy, as shown in Table 5. EVC alone showed moderate diagnostic performance, with diagnostic accuracy ranging from 62% to 76%, sensitivity from 47% to 73%, and specificity from 76% to 90%. A study by Radunski et al. [19], which involved a substantial number of subacute cases of myocarditis, demonstrated the highest diagnostic accuracy among all the studies.

The main findings of each study are summarized in Table 6.

## 4. Discussion

This systematic review of the literature presents findings from a variety of studies that offer valuable insights into the diagnostic capabilities of different CMR imaging methods, notably native T1 mapping, T2 mapping, LGE, and ECV measurement, in the setting of acute myocarditis. Studies indicate that T1 mapping and T2 mapping serve as valuable additions to the original LLC, offering a high level of precision in the diagnosis of acute myocarditis. Moreover, they enable the early detection of myocardial abnormalities and the identification of additional regions of myocardial damage beyond what conventional assessments can reveal [14,22]. This suggests that T2 mapping could potentially replace T2-weighted imaging for detecting myocardial edema, while native T1 mapping could substitute for T1-weighted imaging. Integrating LGE with parametric mapping and ECV enhances the ability to detect different tissue changes such as edema, necrosis, and fibrosis. This improvement in imaging greatly increases the precision of CMR in distinguishing individuals with myocarditis from those who are healthy. Nevertheless, ECV measures may be less precise for identifying active inflammation in the heart muscle because chronic damage accompanied by myocardial fibrosis can lead to elevated ECV values [24]. Table 7 summarizes the characteristics, advantages, limitations, and accuracy of T1 and T2 mapping, LGE, ECV, and a combination of the parameters in diagnosing acute myocarditis.

Native T1 mapping. Notably, Hinojar et al. reported the highest sensitivity (98%) and specificity (100%), indicating exceptional diagnostic performance in detecting acute viral myocarditis. In addition, the study demonstrated that native T1 mapping can distinguish between the acute and convalescent stages of myocarditis [15]. However, it is controversial due to a study by Bohnen et al., which found no significant differences in global native T1 values between patients with and without active myocarditis, which was confirmed by EMB. It is worth noting that the latter study population was limited (n = 31) [24].

Two studies demonstrated that native T1 mapping can detect myocardial injury beyond what is visible on STIR imaging and LGE, indicating its potential as a more sensitive diagnostic tool [14,22]. Ferreira et al. compared T1 mapping with the conventional T2-weighted approach and LGE imaging and found that T1 mapping detected abnormalities in 30% of cases where LGE and T2-weighted sequences failed. In addition, the study showed that native T1 maps, similar to LGE imaging, can identify non-ischemic patterns in acute myocarditis without the requirement of contrast agents [14].

The Jahnke et al. study compared acute myocarditis, non-ST-elevation myocardial infarction (NSTEMI) patients, and a control group and reported that the visual evaluation of T1 maps could potentially differentiate between myocardial infarction, “infarct-like” myocarditis, and healthy controls without the need for quantitative values [17].

Dabir et al. used different approaches to measure native T1 relaxation times, including the complete apical, midventricular, and basal short-axis slice (global); the complete midventricular short-axis slice (mSAX); the midventricular septal wall (ConSept); and the remote myocardium. The global measurement approach provided the overall best diagnostic performance for T1 mapping in acute myocarditis [22].

Huber et al. discovered that myocardial T1 mapping can detect cardiac inflammation, but it is unable to differentiate from idiopathic inflammatory myopathy, which can mimic the clinical manifestations of acute myocarditis. They suggested using skeletal muscle T1 mapping for differentiation [16].

The diagnostic accuracy was lowest in the Radunski et al. study at 69% [19]. This difference could be due to the more subacute clinical presentation of patients in the study population, who underwent CMR at a median of 2 weeks (IQR: 1 to 7 weeks) after onset of symptoms compared to other studies that performed CMR earlier in the acute phase of myocarditis.

T2 mapping. Dabir et al. studied various measurement approaches (global, mSAX, ConSept, remote) and concluded that global measurement is the most effective using T2 mapping. However, all methods demonstrated high diagnostic performance with an AUC above 0.8. This study showed that T1, T2, and ECV values were significantly higher using the global measurement approach in patients with normal standard CMR sequences [22].

Baebler et al. addressed a common limitation of the standard T2 mapping, which averages myocardial T2 over multiple segments and does not consider the focal nature of myocarditis in most cases. They proposed a new approach, using specific parameters to analyze T2. The combination of the highest segmental T2 value (maxT2) and the mean absolute deviation of log-transformed pixel-SD (madSD) was found to be the best discriminator between healthy volunteers and patients, achieving a sensitivity of 81% and a specificity of 83%. Additionally, a multiparametric imaging model, incorporating LGE and feature tracking-derived strain parameters, has been found to further enhance diagnostic accuracy to 94% [13].

The Bohnen et al. study showed that T2 relaxation times can be a highly useful and better parameter than T1 mapping in distinguishing between the acute and convalescent stages of myocarditis [24]. Additionally, several studies showed that T2 mapping is more sensitive for identifying myocardial inflammation in myocarditis than T2-weighted CMR [18,19]. However, when discussing the analysis of visual patterns, Jahnke and colleagues observed that T2 mapping may not be as useful in differentiating between myocardial infarction and “infarct-like” myocarditis compared to T1 mapping and conventional methods, which have this capability [17]. The diagnostic accuracy of T2 mapping did not outperform single standard LLC parameters in the Radunski et al. study, possibly due to the patients’ more subacute clinical presentation, as discussed earlier [19].

Late Gadolinium Enhancement (LGE). Several studies reported that LGE was the most accurate among the original LCC parameters [18,20] and even outperformed the original LLC (88.5% compared to 84.2%) [12]. In a study conducted by Vágó et al., a non-ischemic pattern of LGE with subepicardial and/or midmyocardial involvement proved to be useful in distinguishing between myocarditis, myocardial infarction, and Takotsubo cardiomyopathy in patients presenting with troponin-positive chest pain [21].

Extracellular Volume (ECV). Diagnostic accuracy of ECV may be influenced during the early phase of the disease, when intracellular edema is more common than interstitial edema, and ECV levels may remain within the normal range. Thus, when used alongside LGE, ECV enhanced the diagnostic performance for patients with severe subacute myocarditis compared to the original LLC [19].

Combined approach of CMR parameters. According to different published studies, the diagnostic performance of T2-weighted, EGE, and LGE is 73, 73, and 83 (median AUC, calculated as the average of the sensitivity and specificity), respectively [11]. Alis et al. reported the diagnostic accuracy of edema, hyperemia, LGE, and the LLC (at least two of three components) was 75.7%, 64.2%, 88.5%, and 84.2%, respectively [12]. Using the combined LLC, CMR in the Luetkens et al. study population yielded a sensitivity of 82%, a specificity of 98%, and a diagnostic accuracy of 92% [18].

**Figure 2:**
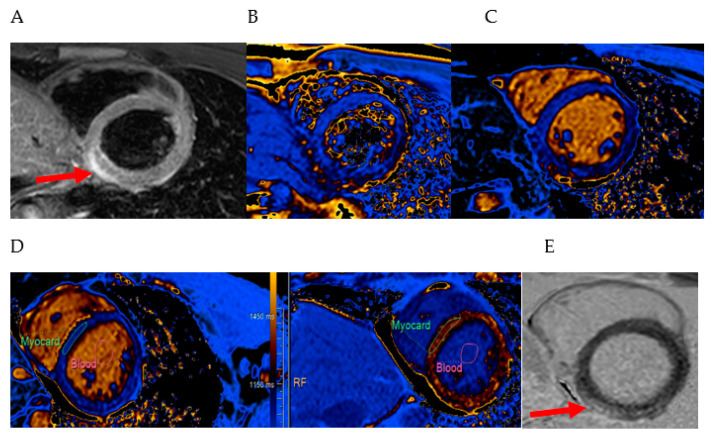
CMR image examples of acute myocarditis, VUH Santaros Klinikos. A 28-year-old male patient with acute myocarditis. (**A**) T2-STIR sequence, red arrow: increased T2 signal intensity in LV inferior wall; (**B**) T2 mapping: increased native T2 to 54 ms (normal value 49 ± 2 ms); (**C**) T1 mapping: increased native T1 to 1048 ms (normal value 969 ± 36 ms); (**D**) ECV: increased ECV to 29% (normal value 24 ± 2%); (**E**) LGE, red arrow: subepicardial contrast material enhancement in the inferior wall of LV. STIR—short tau inversion recovery; LV—left ventricle; ECV—extracellular volume; LGE—late gadolinium enhancement.

However, a strategy combining LGE with other parameters provided the most accurate diagnosis. Hinojar et al. [15] suggested a combined approach using LGE with native T1 mapping, which increased diagnostic accuracy to 87%. The study by Luetkens et al. supported this and reported a diagnostic accuracy of 96%, while also recommending the combination of LGE and T2 mapping for achieving the same level of accuracy [18]. Jahnke et al. found that using LGE with T2-weighted and cine imaging effectively visually distinguishes between NSTEMI, “infarct-like” myocarditis, and healthy controls [17]. Radunski et al. [19] identified acute myocarditis by the presence of LGE or an increased global myocardial ECV ≥ 27% in LGE-negative patients, resulting in a superior accuracy of 90%. In conclusion, the diagnostic performance of CMR could be enhanced when native T1 and T2 relaxation times were combined with LGE. We represent CMR image examples of acute myocarditis in Figure 2.

In the literature, we found systematic literature reviews and meta-analyses performed in previous years from 2017 until 2022. Previous reviews’ conclusions, some from smaller sample sizes, support our findings in the recent literature review. Shaun Khanna et al.’s meta-analysis of 25 studies demonstrated that beyond LGE, acute myocarditis is most reliably differentiated from healthy controls using T1 and T2 mapping (greatest overall effect sizes). The CMR measure of ECV demonstrated a smaller effect size than T1 and T2 mapping [28]. A smaller meta-analysis performed by Zhi Jia et al., which included 400 myocarditis patients and 266 controls, showed that T1 and T2 mapping, including ECV alone, offer comparably good diagnostic performance for the detection of acute myocarditis. They suggested that the reason for the observed mismatch with EMB findings should be further investigated [29]. Juan Xu et al.’s 2020 meta-analysis included only eight studies and concluded that CMR has high sensitivity (94%) and moderate specificity (75%) for viral myocarditis [30]. Pan et al.’s meta-analysis of 867 myocarditis patients and 441 control subjects found that native T1, T2, and ECV mapping provide comparable diagnostic performance to the LLC. Although only native T1 had significantly better sensitivity than the LLC, each technique offers distinct advantages for evaluating and characterizing myocarditis when compared with the LLC [31]. Twenty-two studies were included in the systematic review and meta-analysis performed in 2018 by Kotanidis et al. Novel CMR mapping techniques provide high diagnostic accuracies for diagnosing acute myocarditis and constitute promising successors of the classic elements of the LLC for routine diagnostic protocols [32].

These results emphasize the significance of using multimodal CMR imaging for identifying and describing acute myocarditis. While each technique has its strengths and limitations, their complementary roles in providing comprehensive tissue characterization and diagnostic accuracy highlight the potential for improved patient management and outcomes. This holds significant relevance because myocarditis diagnostic factors became more crucial in the post-COVID-19 era due to reported cases of myocarditis associated with the infection and vaccination efforts [33,34]. The studies showed inconsistent diagnostic accuracy values and thresholds of the parameters. Updated protocols and additional research are essential to establish standard reference ranges for native T1 and T2 relaxation times, given substantial variations among patients’ clinical factors and CMR variability between vendors and machines.

### Limitations

Our review has some limitations. First, we included studies with small sample sizes. CMR was performed at different times from the onset of symptoms. We included one study with a combined population of more subacute cases of myocarditis. Moreover, we did not analyze data on the clinical characteristics of the patients included. In the lack of standardized protocols on T1 and T2 mapping sequences, we did not analyze the impact of the clinical utility and accuracy of T1 and T2 mapping to vendor-specific sequences.

## 5. Conclusions

Novel quantitative tissue markers, such as T1 and T2 mapping, which offer high diagnostic performance, play a crucial role in addressing the diagnostic complexities associated with acute myocarditis. However, it is still early to determine whether mapping can replace some or all the conventional CMR sequences for evaluating myocarditis. Including these parameters in routine clinical practice amplifies the significance of CMR imaging and positions it as a superior alternative to invasive methods, such as EMB, for the characterization of myocardial tissue and the differentiation of various myocardial diseases.

## Figures and Tables

**Table 1:** Characteristics of analyzed studies.

				Patient Baseline Characteristics (Myocarditis Group/Control Group)				CMR Imaging								
No.	Author and Year	Country	Type of Study	Sample Size (n)	Age	Male (%)	Patient Groups	Index Measured Using CMR	CMR Parameters Analyzed	Field Strenght	Reference Standart	CMR Performed, Days after Symptoms Onset	LGE (+) n, (%)	T1 Map n, (%)	T2 Map n, (%)	ECV n, (%)
1	Alis et al., 2020 [12]	Turkey	Retrospective Case-control	n = 68	38.15 ± 10.8/35.12 ± 8.9	68/67	suspected AM (n = 44) control group (n = 24)	LGE, EGEr	Edema, EGE, LGE	1.5 T	Clinical criteria	5.67 ± 2.95	38 (86.4)	N/A	N/A	N/A
2	Baeßler et al., 2015 [23]	Germany	Retrospective Case-control	n = 61	40 ± 15/36 ± 13	81/47	suspected AM (n = 31) control group (n = 30)	maxT2, madSD, maxSD	T2, T2 mapping	1.5 T	Clinical criteria	N/A	N/A	N/A	31 (100)	N/A
3	Baeßler et al., 2017 [13]	Germany	Retrospective Case-control	n = 84	37 ± 14/36 ± 12	73/65	suspected AM (n = 67) control group (n = 17)	T2 mapping, LGE	T2 mapping	1.5 T	Clinical criteria	4.8 ± 4.4	67 (100)	N/A	67 (100)	N/A
4	Bohnen et al., 2015 [24]	Germany	Prospective Cohort	n = 31	48.5 ± 14.5/49.5 ± 12.5	75/80	EMB verified AM (n = 16) EMB negative group (n = 15)	T1 mapping, ECV, T2 mapping	T1 mapping, ECV, T2 mapping	1.5 T	EMB	30 ± 27	N/A	16 (100)	16 (100)	16 (100)
5	Dabir et al., 2019 [22]	Germany	Prospective Case-control	n = 80	38 ± 16.3/36.9 ± 13.5	77/74	suspected AM (n = 50) control group (n = 30)	T1&T2 relaxation time, T2 ratio, EGE ratio, LGE, ECV	T1 mapping, T2 mapping, ECV	1.5 T	Clinical criteria	2.9 ± 2.2	N/A	50 (100)	50 (100)	50 (100)
6	Ferreira et al., 2014 [14]	United Kingdom	Prospective Case-control	n = 110	41 ± 16/41 ± 13	75/74	suspected AM (n = 60) control group (n = 50)	T1 mapping, dark-blood T2, LGE	T1 mapping, dark-blood T2, LGE	1.5 T	Clinical criteria	3.5 ± 2.5	60 (100)	60 (100)	N/A	N/A
7	Hinojar et al., 2015 [15]	United Kingdom	Prospective Case-control	n = 101	48 ± 17/45 ± 15	52/53	suspected AM (n = 61) control group (n = 40)	native T1, post-contrast T1, LGE, T2 signal	native T1, post-contrast T1, LGE, T2 signal	1.5 T, 3 T	Clinical criteria	5 ± 7	N/A	61 (100)	N/A	61 (100)
8	Huber et al., 2018 [16]	France	Retrospective Case-control	n = 60	35 ± 13/47 ± 12	80/82	AM (n = 20) IIM (n = 20) control group (n = 20)	native T1,post-contrast T1, T2, ECV, LGE	native T1,post-contrast T1, T2, ECV, LGE	1.5 T	Clinical criteria	5.18 ± 3.96	20 (100)	20 (100)	20 (100)	20 (100)
9	Jahnke et al., 2023 [17]	Germany	Retrospective Case-control	n = 60	37.5 ± 6.5/40.5 ± 5.5	85/85	NSTEMI (n = 20) infarct-like AM (n = 20) control group (n = 20)	cine, T2w, LGE, T1 maps, T2 maps	T2w, LGE, T1 maps, T2 maps	1.5 T	Clinical criteria	14.5 ± 12.5	20 (100)	20 (100)	20 (100)	N/A
10	Luetkens et al., 2016 [18]	Germany	Prospective case-control	n = 84	44.9 ± 18.7/39.2 ± 17.2	50/60	suspected AM (n = 34) control group (n = 50)	T1, T2 relaxation times, ECV, T2- ratio, LGE, EGE	T1, T2 relaxation times, ECV, T2- ratio, LGE, EGEr	1.5 T	Clinical criteria	2.63 ± 1.93	34 (100)	34 (100)	34 (100)	34 (100)
11	Radunski et al., 2014 [19]	Germany	Retrospective case-control	n = 125	45.5 ± 12.5/37.5 ± 9.5	76/81	suspected AM (n = 104) control group (n = 21)	T2w, EGE, LGE, T2 mapping, native T1, EVC	T1, T2, ECV	1.5 T	Clinical criteria	28 ± 21	104 (100)	104 (100)	104 (100)	104 (100)
12	Schwab et al., 2016 [20]	Germany	Retrospective Case- control	n = 78	34.7 ± 15.2/35.4 ± 13.8	88/89	clinically verified AM (n = 43) control group (n = 35)	T2w, LGE, EGE	T2w, LGE, EGE	1.5 T	Clinical criteria	3 (1–17)	43 (100)	N/A	N/A	N/A
13	Vágó et al., 2020 [21]	Hungary	Retrospective Cohort	N = 250	34 ± 10/49 ±14	88/51	AM (n = 136) MI (n = 55) Takotsubo syndrome (n = 26) control group (n = 20)	LGE, T2 signal	LGE, T2 signal	1.5 T	Clinical criteria	2.7	136 (100)	N/A	N/A	N/A

**Table 2:** Diagnostic performance of T1 mapping in acute myocarditis.

	Sample Size (n)	Sensitivity (%)	Specificity (%)	NPV (%)	PPV (%)	Diagnostic Accuracy (%)	Cut-Off Value (ms)
Dabir et al., 2019 [22]	80	85	90	79	93	87	>980
Ferreira et al., 2014 [14]	110	90	88	88	90	89	>990
Hinojar et al., 2015 [15]	101	98	100	99	100	99	>992
Jahnke et al., 2023 [17]	40	85	87	85	85	80	N/A
Luetkens et al., 2016 [18]	84	85	96	90	94	92	>1000
Radunski et al., 2014 [19]	125	64	90	34	97	69	>1074

**Table 3:** Diagnostic performance of T2 mapping in acute myocarditis.

	Sample Size (n)	Sensitivity (%)	Specificity (%)	NPV (%)	PPV (%)
Baeßler et al., 2015 [23]	61	67	87	N/A	N/A
Baeßler et al., 2017 [13]	84	69	82	40	94
Bohnen et al., 2015 [24]	31	94	60	90	71
Dabir et al., 2019 [22]	80	80	87	74	90
Jahnke et al., 2023 [17]	40	48	63	55	56
Luetkens et al., 2016 [18]	84	79	92	87	87
Radunski et al., 2014 [19]	125	57	89	35	95

**Table 4:** Diagnostic performance of LGE in acute myocarditis.

LGE	Sample Size (n)	Sensitivity (%)	Specificity (%)	NPV (%)	PPV (%)	Diagnostic Accuracy (%)
Alis et al., 2020 [12]	68	86	92	80	95	88.5
Baeßler et al., 2017 [13]	84	52	100	35	100	62
Ferreira et al., 2014 [14]	110	72	97	67	98	81
Hinojar et al., 2015 [15]	101	72	100	79	100	86
Jahnke et al., 2023 [17]	40	92	77	88	78	74
Luetkens et al., 2016 [18]	84	74	100	85	100	89
Radunski et al., 2014 [19]	125	61	100	34	100	67
Schwab et al., 2016 [20]	78	86	100	85	100	92

**Table 5:** Diagnostic performance of ECV in acute/subacute myocarditis.

ECV	Sample Size (n)	Sensitivity (%)	Specificity (%)	NPV (%)	PPV (%)	Diagnostic Accuracy (%)	Cut-Off Value (%)
Dabir et al., 2019 [22]	80	47	88	49	87	62	>31
Luetkens et al., 2016 [18]	84	70	76	79	67	74	>28.8
Radunski et al., 2014 [19]	125	73	90	40	97	76	≥29

**Table 6:** The main findings of the studies.

No.	Author and Year	Key Points
1	Alis et al., 2020 [12]	LGE and/or edema as a sole criterion for the diagnosis of acute myocarditis demonstrated better diagnostic accuracy than the LLC
2	Baeßler et al., 2015 [23]	The proposed cut-off values for maxT2 and madSD in the setting of acute myocarditis allow edema detection with high sensitivity and specificity and, therefore, have the potential to overcome the hurdles of T2 mapping for its integration into clinical routine
3	Baeßler et al., 2017 [13]	A multiparametric CMR imaging model, including the novel T2-mapping-derived parameter madSD, the feature-tracking derived strain parameter, and LGE, yields superior diagnostic sensitivity in suspected acute myocarditis when compared to any imaging parameter alone and to the LLC
4	Bohnen et al., 2015 [24]	T2 mapping seems to be superior when compared with standard CMR parameters, global myocardial T1, and ECV values for assessing the activity of myocarditis in patients with recent-onset heart failure and reduced left ventricular function
5	Dabir et al., 2019 [22]	Native T1 and T2 mapping allow for accurate detection of acute myocarditis irrespective of the measurement approach used
6	Ferreira et al., 2014 [14]	Native T1 mapping can display the typical non-ischemic patterns in acute myocarditis, like LGE imaging, but without the need for contrast agents
7	Hinojar et al., 2015 [15]	The new diagnostic algorithm using native T1 can reliably discriminate between health and disease and determine the clinical disease stage in patients with a clinical diagnosis of myocarditis
8	Huber et al., 2018 [16]	CMR myocardial mapping detects cardiac inflammation in acute viral myocarditis compared to normal myocardium in healthy controls
9	Jahnke et al., 2023 [17]	The conventional approach provided reliable visual discrimination between NSTEMI, myocarditis, and controls
10	Luetkens et al., 2016 [18]	Myocardial T1 and T2 relaxation times were the only parameters of active inflammation/edema that could discriminate between myocarditis patients and control subjects, even at a convalescent stage of the disease
11	Radunski et al., 2014 [19]	In patients with clinical evidence for subacute, severe myocarditis, ECV quantification with LGE imaging significantly improved the diagnostic accuracy of CMR compared with standard LLC
12	Schwab et al., 2016 [20]	Functional and morphological CMR parameters, in addition to tissue characterization, are useful tools in the diagnosis of acute myocarditis
13	Vágó et al., 2020 [21]	CMR performed in the early phase establishes the proper diagnosis in patients with troponin-positive acute chest pain and non-obstructed coronary arteries and provides additional prognostic factors

**Table 7:** Characteristics, advantages, limitations, and accuracy of CMR parameters in diagnosing acute myocarditis.

Parameter	Characteristics	Advantages	Limitations	Diagnostic Accuracy %
T1 mapping	Detection of myocardial edema, inflammation, and diffuse fibrosis	- Quantitative tissue characterization - Non-contrast evaluation - Detection of diffuse fibrosis - Identifies subtle changes in myocardial tissue	- Vendor-specific sequence - No standardized protocols - Breath-holding requirements - Heart rate dependence - Sensitive to motion artifacts	69–99
T2 mapping	- Quantitative evaluation of tissue water content - Diagnosis of myocardial inflammation	- Quantitative tissue characterization - No contrast required	- Vendor-specific sequence - No standardized protocols - Sensitive to motion and susceptibility artifacts - Breath-holding requirements - Heart rate dependence	47–87
LGE	- Detects areas of myocyte necrosis and hyperemia - Delayed imaging: images are taken 10–20 min after gadolinium contrast administration - Areas of fibrosis appear hyperintense compared to normal myocardium	- Distinguishing between ischemic and non-ischemic etiology of heart diseases - Accurately identifies areas of focal fibrosis	- Qualitative or semiquantitative data - Requires contrast agent - Incomplete myocardium nulling - Insensitive to detecting diffuse interstitial fibrosis - Sensitive to motion artifacts - Does not differentiate well between acute and chronic myocardial injury - Contraindicated in patients with severe renal dysfunction - Limited spatial resolution	62–92
ECV	- Calculated using native and post-contrast T1 mapping - Used to assess the cellular and extracellular interstitial matrix compartments, represented as volume proportions	- Quantitative measurement	- Requires contrast agent - Technical variability - No standardized protocols - Influence of hematocrit - Sensitive to motion artifacts	62–76
Combination of the parameters	T1 mapping, T2 mapping, LGE	- Detailed evaluation of myocardial tissue - Enhanced diagnostic accuracy	- Requires advanced imaging protocols and expertise - Increased scan time - Motion artifacts - Standardization variability - Requires contrast agent	87–96

## Data Availability

No new data were created.

## Supplementary Materials

The following supporting information can be downloaded at: https://www.mdpi.com/article/10.3390/medicina60071162/s1, Table S1: QUADAS-2.

## References

[1] Sagar S., Liu P.P., Cooper L.T. (2012). Myocarditis. Lancet.

[2] Ammirati E., Veronese G., Bottiroli M., Wang D.W., Cipriani M., Garascia A., Pedrotti P., Adler E.D., Frigerio M. (2021). Update on acute myocarditis. Trends Cardiovasc. Med..

[3] Fung G., Luo H., Qiu Y., Yang D., McManus B. (2016). Myocarditis. Circ. Res..

[4] Leone O., Pieroni M., Rapezzi C., Olivotto I. (2019). The spectrum of myocarditis: From pathology to the clinics. Virchows Arch..

[5] Caforio A.L.P., Pankuweit S., Arbustini E., Basso C., Gimeno-Blanes J., Felix S.B., Fu M., Heliö T., Heymans S., Jahns R. (2013). Current state of knowledge on aetiology, diagnosis, management, and therapy of myocarditis: A position statement of the European Society of Cardiology Working Group on Myocardial and Pericardial Diseases. Eur. Heart J..

[6] Dai H., Lotan D., Abu Much A., Younis A., Lu Y., Bragazzi N.L., Wu J. (2020). Global, regional, and national burden of myocarditis and cardiomyopathy, 1990–2017. medRxiv.

[7] Mistrulli R., Ferrera A., Muthukkattil M.L., Volpe M., Barbato E., Battistoni A. (2023). SARS-CoV-2 Related Myocarditis: What We Know So Far. J. Clin. Med..

[8] Lewis A.J.M., Burrage M.K., Ferreira V.M. (2020). Cardiovascular magnetic resonance imaging for inflammatory heart diseases. Cardiovasc. Diagn. Ther..

[9] Friedrich M.G., Sechtem U., Schulz-Menger J., Holmvang G., Alakija P., Cooper L.T., White J.A., Abdel-Aty H., Gutberlet M., Prasad S. (2009). Cardiovascular Magnetic Resonance in Myocarditis: A JACC White Paper. J. Am. Coll. Cardiol..

[10] Carrabba N., Amico M.A., Guaricci A.I., Carella M.C., Maestrini V., Monosilio S., Pedrotti P., Ricci F., Monti L., Figliozzi S. (2024). CMR Mapping: The 4th-Era Revolution in Cardiac Imaging. J. Clin. Med..

[11] Ferreira V.M., Schulz-Menger J., Holmvang G., Kramer C.M., Carbone I., Sechtem U., Kindermann I., Gutberlet M., Cooper L.T., Liu P. (2018). Cardiovascular Magnetic Resonance in Nonischemic Myocardial Inflammation: Expert Recommendations. J. Am. Coll. Cardiol..

[12] Alis D., Güler A., Aşmakutlu O., Uygur B., Ördekçi S. (2020). Diagnostic values of edema-sensitive T2-weighted imaging, TSE T1-weighted early contrast-enhanced imaging, late gadolinium enhancement, and the Lake Louise criteria in assessing acute myocarditis: A single-center cardiac magnetic resonance study. Turk Kardiyol. Dern. Ars..

[13] Baeßler B., Treutlein M., Schaarschmidt F., Stehning C., Schnackenburg B., Michels G., Maintz D., Bunck A.C. (2016). A novel multiparametric imaging approach to acute myocarditis using T2-mapping and CMR feature tracking. J. Cardiovasc. Magn. Reson..

[14] Ferreira V.M., Piechnik S.K., Dall’Armellina E., Karamitsos T.D., Francis J.M., Ntusi N., Holloway C., Choudhury R.P., Kardos A., Robson M.D. (2014). Native T1-mapping detects the location, extent and patterns of acute myocarditis without the need for gadolinium contrast agents. J. Cardiovasc. Magn. Reson..

[15] Hinojar R., Foote L., Arroyo Ucar E., Jackson T., Jabbour A., Yu C.Y., McCrohon J., Higgins D.M., Carr-White G., Mayr M. (2015). Native T1 in discrimination of acute and convalescent stages in patients with clinical diagnosis of myocarditis: A proposed diagnostic algorithm using CMR. JACC Cardiovasc. Imaging.

[16] Huber A.T., Bravetti M., Lamy J., Bacoyannis T., Roux C., de Cesare A., Rigolet A., Benveniste O., Allenbach Y., Kerneis M. (2018). Non-invasive differentiation of idiopathic inflammatory myopathy with cardiac involvement from acute viral myocarditis using cardiovascular magnetic resonance imaging T1 and T2 mapping. J. Cardiovasc. Magn. Reson..

[17] Jahnke C., Sinn M., Hot A., Cavus E., Erley J., Schneider J., Chevalier C., Bohnen S., Radunski U., Meyer M. (2023). Differentiation of acute non-ST elevation myocardial infarction and acute infarct-like myocarditis by visual pattern analysis: A head-to-head comparison of different cardiac MR techniques. Eur. Radiol..

[18] Luetkens J.A., Homsi R., Sprinkart A.M., Doerner J., Dabir D., Kuetting D.L., Block W., Andrié R., Stehning C., Fimmers R. (2016). Incremental value of quantitative CMR including parametric mapping for the diagnosis of acute myocarditis. Eur. Heart J.—Cardiovasc. Imaging.

[19] Radunski U.K., Lund G.K., Stehning C., Schnackenburg B., Bohnen S., Adam G., Blankenberg S., Muellerleile K. (2014). CMR in patients with severe myocarditis: Diagnostic value of quantitative tissue markers including extracellular volume imaging. JACC Cardiovasc. Imaging.

[20] Schwab J., Rogg H.-J., Pauschinger M., Fessele K., Bareiter T., Bär I., Loose R. (2015). Functional and Morphological Parameters with Tissue Characterization of Cardiovascular Magnetic Imaging in Clinically Verified “Infarct-like Myocarditis”. Rofo.

[21] Vágó H., Szabó L., Dohy Z., Czimbalmos C., Tóth A., Suhai F.I., Bárczi G., Gyarmathy V.A., Becker D., Merkely B. (2020). Early cardiac magnetic resonance imaging in troponin-positive acute chest pain and non-obstructed coronary arteries. Heart.

[22] Dabir D., Vollbrecht T.M., Luetkens J.A., Kuetting D.L.R., Isaak A., Feisst A., Fimmers R., Sprinkart A.M., Schild H.H., Thomas D. (2019). Multiparametric cardiovascular magnetic resonance imaging in acute myocarditis: A comparison of different measurement approaches. J. Cardiovasc. Magn. Reson..

[23] Baeßler B., Schaarschmidt F., Dick A., Stehning C., Schnackenburg B., Michels G., Maintz D., Bunck A.C. (2015). Mapping tissue inhomogeneity in acute myocarditis: A novel analytical approach to quantitative myocardial edema imaging by T2-mapping. J. Cardiovasc. Magn. Reson..

[24] Bohnen S., Radunski U.K., Lund G.K., Kandolf R., Stehning C., Schnackenburg B., Adam G., Blankenberg S., Muellerleile K. (2015). Performance of t1 and t2 mapping cardiovascular magnetic resonance to detect active myocarditis in patients with recent-onset heart failure. Circ. Cardiovasc. Imaging.

[25] Tijmes F.S., Thavendiranathan P., Udell J.A., Seidman M.A., Hanneman K. (2021). Cardiac MRI Assessment of Nonischemic Myocardial Inflammation: State of the Art Review and Update on Myocarditis Associated with COVID-19 Vaccination. Radiol. Cardiothorac. Imaging.

[26] Friedrich M.G. (2008). Tissue characterization of acute myocardial infarction and myocarditis by cardiac magnetic resonance. JACC Cardiovasc. Imaging.

[27] Mavrogeni S., Apostolou D., Argyriou P., Velitsista S., Papa L., Efentakis S., Vernardos E., Kanoupaki M., Kanoupakis G., Manginas A. (2017). T1 and T2 Mapping in Cardiology: “Mapping the Obscure Object of Desire”. Cardiology.

[28] Khanna S., Amarasekera A.T., Li C., Bhat A., Chen H.H., Gan G.C., Ugander M., Tan T.C. (2022). The utility of cardiac magnetic resonance imaging in the diagnosis of adult patients with acute myocarditis: A systematic review and meta-analysis. Int. J. Cardiol..

[29] Jia Z., Wang L., Jia Y., Liu J., Zhao H., Huo L., Zheng B. (2021). Detection of acute myocarditis using T1 and T2 mapping cardiovascular magnetic resonance: A systematic review and meta-analysis. J. Appl. Clin. Med. Phys..

[30] Xu J., Xu Y. (2020). Meta-analysis of the Value of Cardiac Nuclear Magnetic Resonance in the Diagnosis of Viral Myocarditis. J. Coll. Physicians Surg. Pak..

[31] Pan J.A., Lee Y.J., Salerno M. (2018). Diagnostic Performance of Extracellular Volume, Native T1, and T2 Mapping Versus Lake Louise Criteria by Cardiac Magnetic Resonance for Detection of Acute Myocarditis. Circ. Cardiovasc. Imaging.

[32] Kotanidis C.P., Bazmpani M.-A., Haidich A.-B., Karvounis C., Antoniades C., Karamitsos T.D. (2018). Diagnostic Accuracy of Cardiovascular Magnetic Resonance in Acute Myocarditis: A Systematic Review and Meta-Analysis. JACC Cardiovasc. Imaging.

[33] Zinkovsky D., Sood M.R., Zinkovsky D., Sood M.R. (2023). The Evaluation of Myocarditis in the Post-Covid-19 Era: Pearls and Perils for the Clinician. Pericarditis-Diagnosis and Management Challenges.

[34] Militaru S., Mihu A., Genunche-Dumitrescu A.V., Neagoe C.D., Avramescu T.E., Istratoaie O., Gheonea I.-A., Militaru C. (2023). Multimodality Cardiac Imaging in COVID-19 Infection. Medicina.

