# Supplementary material for: Diagnostic Value of Cardiovascular Magnetic Resonance T1 and T2 Mapping in Acute Myocarditis: A Systematic Literature Review
Source: Medicina (Kaunas). 2024 Jul 18;60(7):1162. doi: 10.3390/medicina60071162 (PMC11279077; doi:10.3390/medicina60071162)
Supplement: Supplementary file 1 [file medicina-60-01162-s001.zip › medicina-3043894-supplementary.pdf]

		Risk of bias				Applicability concerns		
		Patient Selection	Index Test	Reference Standart	Flow and Timing	Patient Selection	Index Test	Reference Standart
1	Alis et al., 2020	High	Low	Low	Low	Low	Low	Low
2	Baeßler et al., 2015	Low	Unclear	Low	Low	Low	Low	Low
3	Baeßler et al., 2017	Low	Low	Low	Low	Low	Low	Low
4	Bohnen et al., 2015	Low	Low	Low	Low	Low	Low	Low
5	Dabir et al., 2019	Low	Unclear	Low	Low	Low	Low	Low
6	Ferreira et al., 2014	Low	Unclear	Low	Low	Low	Low	Low
7	Hinojar et al., 2015	Low	Unclear	Low	Low	Low	Low	Low
8	Huber et al., 2018	Low	Unclear	Low	Low	Low	Low	Low
9	Jahnke et al., 2023	High	Low	Low	Low	Low	Low	Low
10	Luetkens et al., 2016	High	Low	Low	Low	Low	Low	Low
11	Radunski et al., 2014	Low	Low	Unclear	Low	Low	Low	Low
12	Schwab et al., 2016	Low	Unclear	Low	Low	Low	Low	Low
13	Vágó et al., 2020	Low	Unclear	Low	Low	Low	Low	Low

Table S1. QUADAS-2.
